# Evaluating Graph-Centrality Context for Station-Level Correction of CAMS Air Quality Reanalysis in Lombardy from 2018 to 2021

**DOI:** 10.3390/s26185844

**Published:** 2026-09-15

**Authors:** Alexandru Munteanu, Cosmin Bonchiș, Gerardo López Saldaña

**Affiliations:** 1 Faculty of Computer Science, West University of Timișoara, 300223 Timișoara, Romania; cosmin.bonchis@e-uvt.ro; 2 Assimila Ltd., Reading Enterprise Centre, Whiteknights Rd, Reading RG6 6BU, UK; gerardo.lopezsaldana@assimila.eu

**Keywords:** graph theory, centrality, GTWR, XGBoost, RF-STK, CAMS, ARPA Lombardia

## Abstract

Monitoring air quality is critical for understanding pollution impacts on public health. The Copernicus Atmosphere Monitoring Service (CAMS) provides estimates of nitrogen oxides (NO_*x*_), nitrogen dioxide (NO_2_), fine particulate matter (PM_2.5_) and inhalable coarse particles (PM_10_), but its spatial resolution may limit accuracy at individual urban stations. This study introduces a graph centrality-based framework for station-level correction of CAMS European reanalysis data using in situ observations from ARPA Lombardia (2018 to 2021). Stations are modelled as nodes in a multiplex graph, with spatial influence quantified through six centrality measures including topology-based and context-aware variants such as the Adapted PageRank Algorithm (APA) and Eigendata. The centrality features are integrated with Geo-Temporally Weighted Regression (GTWR), Extreme Gradient Boosting (XGBoost) and Random Forest with Spatio-Temporal Kriging (RF-STK), evaluated under station, temporal and combined holdout strategies. Under station holdout, XGBoost achieves an $R^{2}$ of up to 0.66 for NO_2_, while RF-STK can exceed this when residual spatial kriging is active. Adding OpenStreetMap (OSM)-context APA centrality yields pollutant-dependent changes: gains for NO_*x*_ and PM_2.5_, mixed results for NO_2_, and a temporal improvement for PM_10_ with RF-STK. Results confirm that graph-theoretic structural context complements station-level CAMS correction but is not a general surrogate for pollutant dispersion.

## 1. Introduction

Air quality monitoring has been an important step towards analysing the effects pollution emissions have on the environment, as well as on the general population’s health. The negative impact of common air pollutants on the general health of individuals has been widely studied [1,2]. The region of Lombardy, Italy, ranks among the top polluted regions within Europe [3]. This aspect can be attributed to multiple factors, starting with the geographical characteristics of the region, where cities are located mainly along the Po river basin, being also surrounded by mountains, limiting air mass exchange [1]. Meteorological conditions in Lombardy such as low wind speeds [4] can favour the accumulation of pollutants closer to the ground. In addition, industrial activities carried out in the region can contribute to pollution [5,6].

Reducing pollution has been a long-term concern; therefore, policymakers from both a global and European context have undertaken efforts in order to develop directives and standards [7]. The European Commission (EC) has proposed a set of regulations [8] which are aligned with the World Health Organisation (WHO) guidelines towards meeting a goal of zero emissions. Careful monitoring and analysis of daily pollution emissions is an important part of ensuring that the goals highlighted within these directives are met.

Supporting this effort, the EC established the Copernicus Atmosphere Monitoring Service (CAMS) in 2014 [9]. One of its primary purposes is to deliver valuable global information regarding atmospheric composition on a regular basis. To provide accurate information, CAMS utilises a combination of atmospheric model output, ground-based observations and data assimilation techniques developed by its implementer, the European Centre for Medium-Range Weather Forecasts (ECMWF). One of the products provided by CAMS constitutes an annual air quality reanalysis for Europe which is comprised of an ensemble of nine data assimilation systems for air quality. This product combines model data with observations provided by the European Environment Agency (EEA) [10].

**Figure 1:**
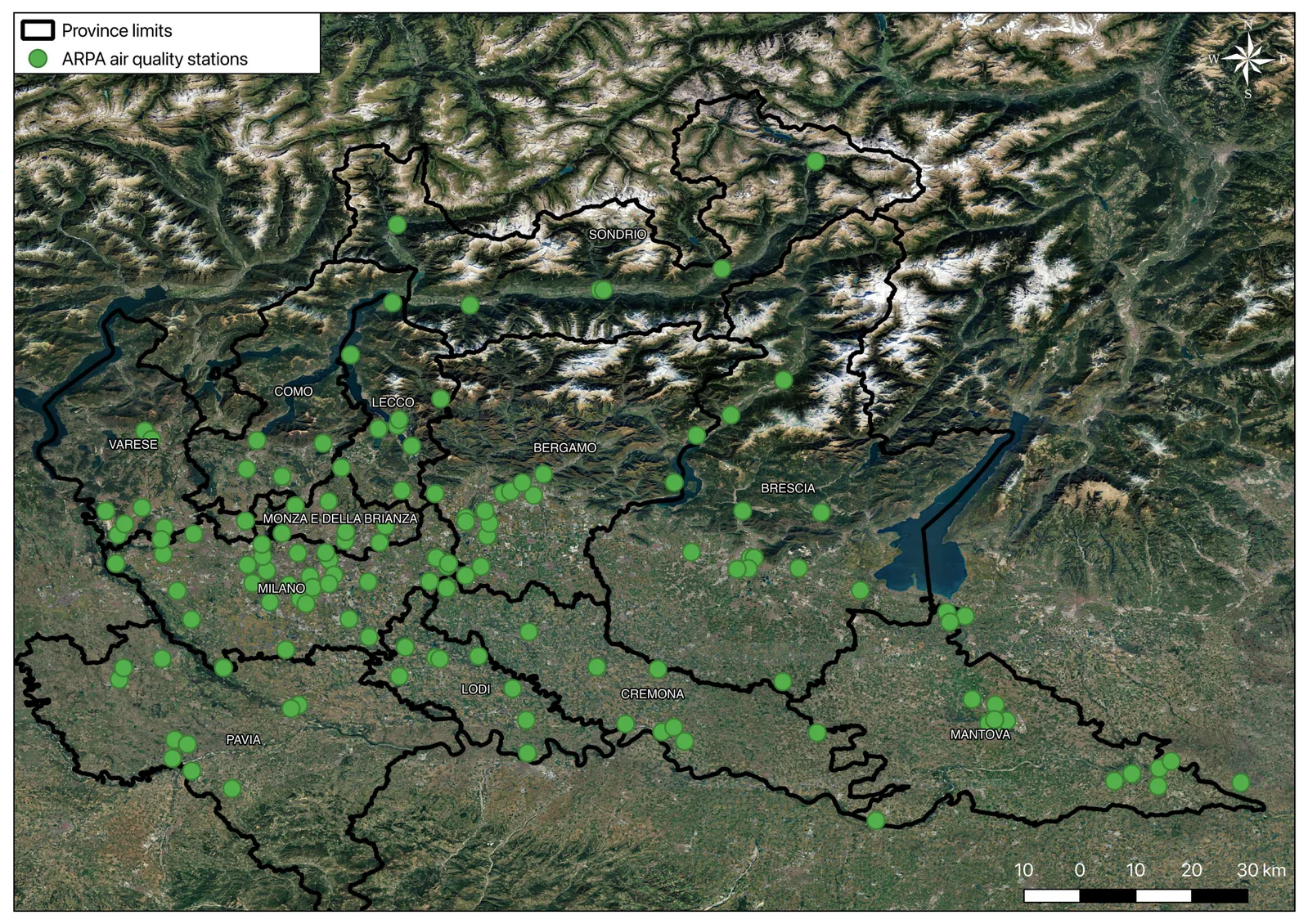
ARPA Lombardia air quality measurement stations used in this study. The map shows the monitoring network for the 2018 to 2021 analysis period together with the limits of each province within the region of Lombardy, Italy. Blue lines denote provincial boundaries; black markers denote monitoring stations.

The air quality and weather monitoring service, ARPA Lombardia (Agenzia Regionale per la Protezione dell’Ambiente Lombardia), described in [4,11], provides valuable insights regarding a range of pollutants. This robust network of air quality stations, strategically distributed throughout the Lombardy region, has been operating for 56 years, consistently providing data at hourly or daily intervals. As of 2026, there are a total of 490 air quality stations, actively monitoring NO_*x*_, NO_2_, PM_2.5_, PM_10_, black carbon (BC), O_3_, CO, SO_2_, benzene (C_6_H_6_), benzo[a]pyrene (C_20_H_12_), NH_3_, Cd, Ni, As and Pb pollutants [4]. The network of ground-level air quality monitoring stations is illustrated in Figure 1.

Studies on the interaction of different pollution emissions in the Po river basin have highlighted their complex, non-linear behaviour [12,13,14]. This distinctive feature strongly motivates further investigation of emissions in the region, while also underscoring the difficulty of developing models that can accurately represent such relationships.

Combining model fields with in situ observations has a substantial non-ML literature, including Bayesian melding [15], external-drift kriging [16], European model observation assimilation intercomparisons [17], statistical downscaling [18,19], and land-use regression [20,21,22], alongside more recent Bayesian space-time fusion [23], geostatistical hybrids with satellite and dispersion-model covariates [24], global atmospheric reanalysis assimilation [25], and machine learning downscaling of chemistry-transport-model output [26]. These approaches establish that model-monitor fusion can correct systematic discrepancies, while also showing that validation must respect spatial and temporal dependence.

The research gap addressed in this paper is narrower: whether a target-independent structural feature derived from the air quality monitoring station graph, specifically OSM-context graph centrality, adds reproducible information beyond CAMS, location and time under spatially and temporally blocked evaluation.

From a graph-theoretic standpoint, the network of air quality monitoring stations operated by ARPA Lombardia can be represented as a multiplex graph. In a multiplex network, each layer is a separate graph that captures a particular type of interaction among the same set of nodes. By considering multiple such layers together, one can model different attributes or features associated with the nodes in the network. In this study, node attributes are derived from OpenStreetMap (OSM) building counts within a configurable spatial radius around each monitoring station, providing a context-aware representation of urban structure that remains entirely independent of pollutant measurements and therefore free from target leakage.

The primary objective of the research conducted in this paper is to evaluate whether the structural intelligence of air quality station networks, captured through graph-theoretical centrality, can refine station-level agreement between regional air quality reanalysis and in situ observations. Building upon the framework established in [27], we integrate graph theory concepts with geo-temporally weighted regression and machine learning to address the following questions:

1. How can graph-theoretical centrality measurements be integrated with CAMS products and in situ measurements under blocked spatial and temporal validation?
2. To what extent do context-aware centrality measurements, specifically Adapted PageRank Algorithm (APA/APAM) [28] or Eigendata [29], provide complementary information relative to their traditional, purely topological variants such as PageRank [30] or Eigenvector [31], when the context vector is built from OSM urban structure rather than pollutant measurements?
3. Which modelling approach between local statistical regression (GTWR [32]) or global non-linear machine learning (XGBoost [33] or Random Forest with Spatio-Temporal Kriging [34]) is better suited for station-level CAMS correction in Lombardy when enriched with topological features?

## 2. Related Work

Leveraging graph theory concepts such as connectivity and centrality to model geospatial information as networks has proven valuable across diverse use cases. These range from general urban network applications for tourism [35] and evaluating regional city centrality [36], to highly specific scenarios. Examples include mapping coastal sediment pathways [37], optimizing pedestrian walkways to minimize PM_2.5_ exposure [38], modelling bicycle routes [39], analysing spatio-temporal air pollution factors [40] and designing new centrality metrics to assess node vulnerability to pollution emissions [41]. Our study differentiates itself by introducing a framework that combines both topology-based and context-aware centrality with machine learning to refine coarse-resolution atmospheric data, a methodology validated through evaluating multiple graph configurations.

### 2.1. Centrality Measurements

This subsection reviews the centrality measures used in our framework such as Betweenness [42], Eigenvector [31] and PageRank [30] with an emphasis on urban-context variants that incorporate georeferenced structural data together with network topology.

Betweenness centrality [42] ranks nodes by their frequency on shortest paths between other node pairs. Current-Flow Betweenness (CFB) [43] extends the concept of paths to an electrical network model, where edges represent resistors and centrality is determined by the total fraction of current that flows through a node when a unit of current is sent between all possible pairs of source and target nodes [43].

To address the computational cost of Current Flow Betweenness [43] in large urban networks, Ref. [44] proposed Resized Current Flow Betweenness (RCFB), which estimates pedestrian or vehicular flow from both topology and urban edge capacities (e.g., road width or pollution sensitivity). RCFB redefines edge weights as conductances and computes:

(1) $$C_{RCFB}\left(n\right)=\frac{1}{\left(N-1)(N-2\right)}\sum_{s,t\in S}I_{n}\left(s,t\right)$$

where $I_{n}\left(s,t\right)$ is the unit current through node *n* for source sink pair (s,t), weighted by the urban capacity of adjacent edges. For monitoring networks such as ARPA Lombardia, RCFB can identify stations that mediate broader connectivity rather than only shortest-path dominance, which is relevant when pollutant influence disperses gradually. Empirical results on the Murcia (Spain) street network confirm that incorporating commercial-activity counts (shop and service premises used as urban context) alters centrality rankings beyond topology alone [44].

Eigenvector centrality [31] assigns higher scores to nodes connected to other highly central nodes, capturing transitive influence across the network. Because purely topological eigenvector scores offer limited discrimination in urban networks with narrow degree distributions, Ref. [29] introduced Eigendata centrality, which weights the adjacency matrix by contextual node attributes *D*:

(2) $$W_{n,m}=A_{n,m}\cdot \sqrt{D(n)\cdot D(m)}$$

The resulting centrality is the principal eigenvector of *W*, combining structural connectivity with functional urban density. Empirical results on the Murcia network show that this weighting yields more discriminative rankings than topology alone when commercial activity data are incorporated [29].

PageRank [30] ranks nodes through a random-walk process with stochastic jumps across the network. The Adapted PageRank Algorithm (APA) [28] extends this idea by combining topological transitions with georeferenced urban data, allowing jumps proportional to contextual node attributes rather than uniform over the network. APA constructs a transition matrix as a linear combination of the topological matrix $A^{*}$ and a data-dependent matrix *V*:

(3) $$M_{APA}=\left(1-\alpha \right)A^{*}+\alpha V$$

where α∈[0,1] controls the influence of urban context. The Adapted PageRank Algorithm Modified (APAM) localises the data matrix *V* to each node’s neighbourhood, reflecting the spatially local nature of urban interactions; the same parameter α balances topological structure against contextual magnitude [28]. Both variants align more closely with observed pedestrian and vehicular flows than topology-only PageRank when contextual data are included, with APAM performing better on locally concentrated activity patterns.

**Figure 2:**
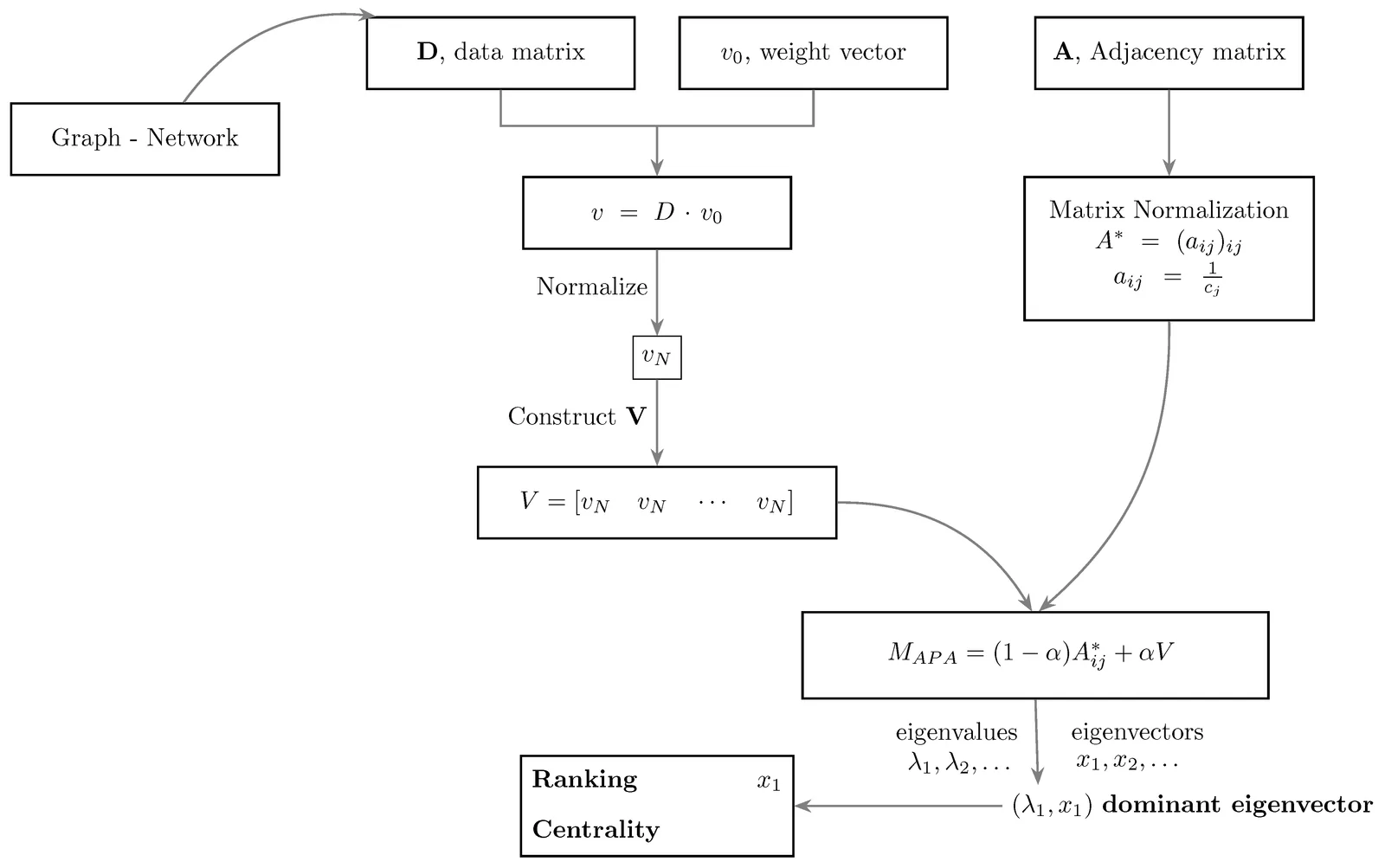
Adapted PageRank Algorithm (APA), adapted from [28]. The unweighted graph defines the row-normalized topological transition matrix $A^{*}$, normalized OSM building counts define the context matrix *V*, and α controls their contribution to the stationary centrality vector. Arrows show the construction sequence from the graph and context vector through *V* and $M_{APA}$ to the centrality ranking.

Figure 2 summarises the APA workflow: contextual attributes are encoded in matrix *D*, expanded into *V*, and combined with the normalised adjacency matrix to form $M_{APA}$, whose dominant eigenvector yields the final centrality ranking.

Centrality-based analytical methods are extensively used to evaluate the importance of nodes within a communication graph based on correlation coefficient matrices. In connection to our study, the authors of [45] investigated the impact of spatial centrality on PM_2.5_ concentrations in urban areas. In the study conducted by [45], high-resolution PM_2.5_ data were gathered using mobile monitoring methods to investigate the spatial-temporal heterogeneity of the relationship between the built environment and on-road PM_2.5_ levels during rush hours. The study used the betweenness centrality measurement in order to assess the impacts of PM_2.5_ concentrations.

Despite recent advancements, which have seen additional analysis employing topological centrality for meteorology or air pollution on a larger scale, our research aims to apply such methodologies, concepts and ideas within a regional area where fixed air quality monitoring stations are present. This approach aims to uncover correlations between in situ measurements and regional reanalysis data.

### 2.2. Geographically Weighted Regression and Geo-Temporally Weighted Regression

Using Geographically Weighted Regression [46,47] (GWR), as well as its multi-scale variant (MGWR) [48], for analysing spatially varying relationships has proven effective in producing global estimates of particulate matter by integrating multi-modal data [49], tracking the impact of spatio-temporal differences in spatial structures of carbon emissions [50] and predicting spatial distributions of PM_2.5_ concentrations in urban areas [51].

GWR [46,47] calibrates a separate local regression at each observation by weighting neighbours through a spatial kernel; bandwidth is typically selected via the Akaike information criterion (AIC) [52] or cross-validation [53,54]. The positive results of merging centrality measurements with GWR have been highlighted for a couple of use cases such as: analysing the impacts of land use and population density towards seasonal surface water quality [55], exploring the driving force contributing towards urbanization [56], modelling annual average daily traffic within a national network using a modified centrality measure [57] or analysing the relationship between PM_2.5_ and built-up areas by integrating betweenness centrality and GWR [58].

While GWR assumes stationarity over time, GTWR [32] extends the kernel to joint spatio-temporal neighbourhoods. Given the seasonality of the air quality measurements in this study, we adopt GTWR, in which spatial distance $d_{ij}$ is replaced by a spatio-temporal distance $d_{ij}^{ST}$:

(4) $${\left(d_{ij}^{ST}\right)}^{2}=\lambda \left[{\left(x_{i}-x_{j}\right)}^{2}+{\left(y_{i}-y_{j}\right)}^{2}\right]+\mu {\left(t_{i}-t_{j}\right)}^{2}$$

where x,y represent the spatial coordinates, *t* is the time coordinate and λ and μ are scale factors used to balance the different units of space and time.

GTWR [32] has been effectively used for estimating ground-level values of PM_2.5_ concentrations from MODIS Aerosol Optical Depth (AOD) products by [59] or analysing the impact of spatio-temporal characteristics of urban agglomeration on carbon emissions [50].

### 2.3. Machine Learning and Non-Linear Modelling

Although GTWR provides a robust statistical framework, the non-linear response of pollutants such as NO_*x*_, NO_2_, PM_2.5_ or PM_10_ to emission changes in the Po basin suggests the need for advanced algorithmic approaches that are capable of capturing these non-linear interactions [12,13,14]. Machine learning models have garnered popularity due to their ability to model complex dependencies without strict distributional assumptions. We have chosen to employ XGBoost and RF-STK due to their proven performance in predicting pollution emissions as pointed out in several studies [34,60,61,62].

Extreme Gradient Boosting (XGBoost) [33] has been developed as an ensemble learning method, being previously used in similar use cases involving urban contexts such as predicting PM_10_, PM_2.5_ and CO emissions in Macau [60], or forecasting PM_2.5_ emissions in Shanghai [63]. By utilizing a gradient boosting framework that optimizes a differentiable loss function, XGBoost has the potential to handle high-dimensional datasets, such as CAMS reanalysis products, and it has been shown to outperform traditional linear models in capturing localised pollution [61].

Similarly, a Random Forest [64] variant combined with Spatio-Temporal Kriging [65] namely RF-STK was introduced by [34] with the scope of estimating daily ambient NO_2_ emissions. This variant offers a hybrid approach by combining the non-linear predictive capabilities of classical Random Forests with the spatial interpolation features of kriging. RF-STK is particularly useful for addressing residuals that exhibit both global non-linearities and local spatio-temporal autocorrelations [34]. Furthermore, studies such as those performed by [62] have successfully employed RF-STK for predicting daily SO_2_ exposure on national levels using ground-based measurements.

The remainder of this paper is structured as follows. Section 3 describes the study area, data sources, preprocessing, graph construction, centrality measures, models and validation design. Section 4 presents the findings on node influence and model performance under blocked holdouts. Section 5 examines the implications of these results, and finally, Section 6 summarizes our main findings and contributions.

## 3. Materials and Methods

This study focuses on the Lombardy region of Italy, utilizing data from a temporal window from 2018 to 2021 in order to capture consistent seasonal and spatial patterns. Lombardy spans dense urban and industrial areas, agricultural plains and Alpine terrain. The enclosed Po Valley setting and restricted ventilation motivate local evaluation of CAMS against the ARPA network. Elevation, topography and meteorological variables (wind, boundary-layer height, atmospheric stability) are not included as predictors in the present design; their omission is discussed as a limitation in Section 5.

### 3.1. CAMS European Air-Quality Reanalysis

**Figure 3:**
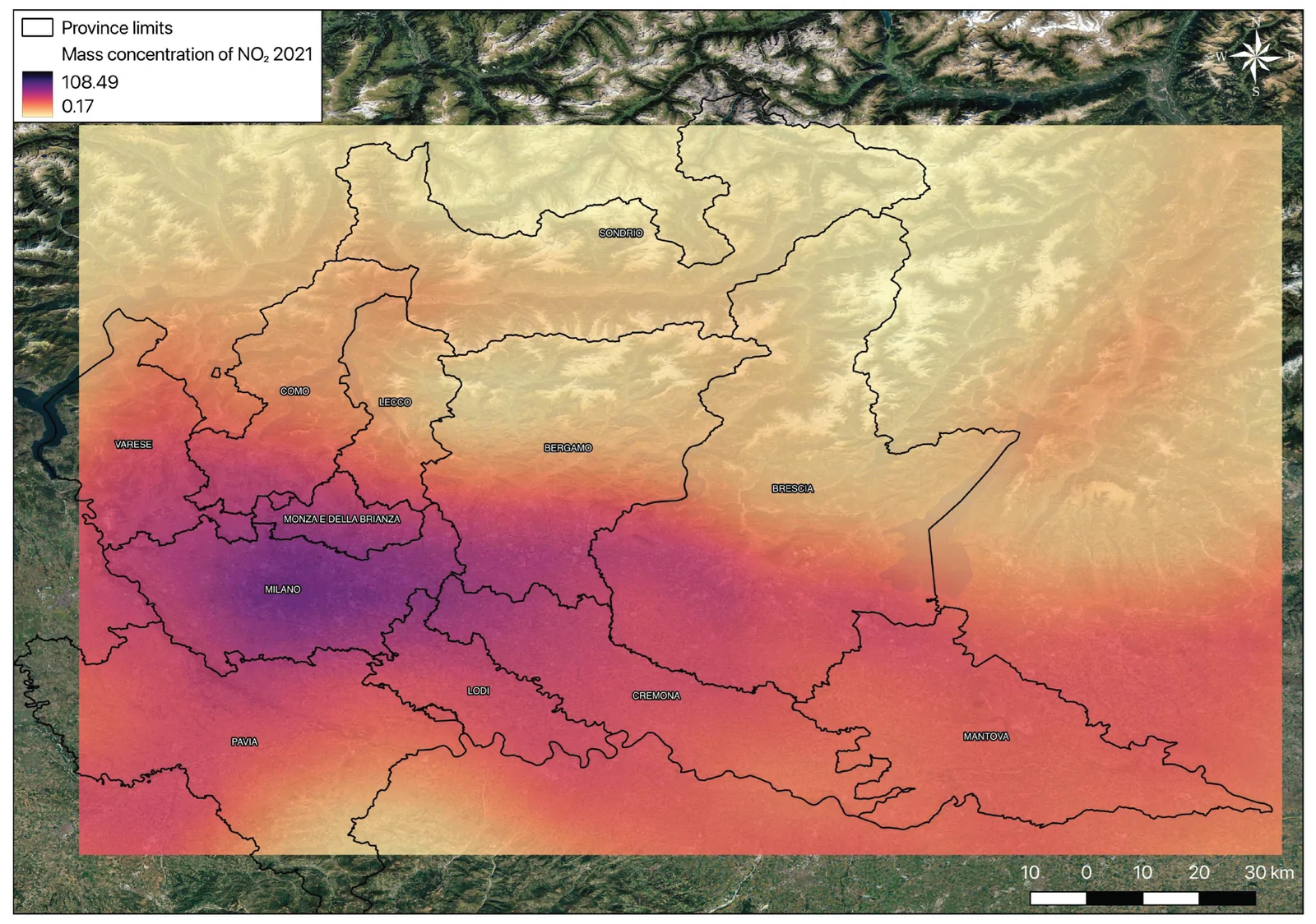
CAMS European Reanalysis data for mass concentrations of NO_2_ in 2021 over Lombardy (μg m^−3^).

The Copernicus Atmosphere Monitoring Service (CAMS) provides the explanatory data for the fusion process. We utilize the CAMS annual air quality reanalysis for Europe (https://ads.atmosphere.copernicus.eu/datasets/cams-europe-air-quality-reanalyses, accessed on 22 May 2026), which represents an ensemble of nine distinct data assimilation systems that combine numerical modelling with European Environment Agency (EEA) observations [9,10]. The gridded products have a 0.1° × 0.1° horizontal resolution, with hourly temporal resolution. Figure 3 shows CAMS European Reanalysis data for mass concentrations of NO_2_ for 2021. CAMS is therefore an assimilated atmospheric-model ensemble rather than a remote-sensing image product.

The grid values have been coregistered with the ARPA station locations through bilinear interpolation. Hourly CAMS grids are first averaged to daily means and then interpolated to each station coordinate. This coregistration process generates the cams_value feature, which is the internal model-feature name for the daily CAMS concentration at each station after bilinear interpolation, expressed in μg m^−3^.

Because the CAMS European reanalysis may assimilate EEA background stations, and ARPA stations report into the EEA network, we position-matched ARPA stations to EEA metadata within 500 m. Of the study stations, 3 of 84 NO_*x*_ stations, 38 of 85 NO_2_ stations, 25 of 64 PM_10_ stations and 14 of 35 PM_2.5_ stations matched assimilation-eligible background sites. Matched traffic or industrial sites were classified as known never assimilated; unmatched sites were not assumed independent. No NO_*x*_ station qualified for the known-never-assimilated subset, so that sensitivity is reported only where the subset is non-empty (notably temporal PM_10_). This dependence makes the analysis an evaluation of station-level correction rather than validation against a fully external network.

### 3.2. ARPA Lombardia In Situ Measurements

The in situ measurements are sourced from the ARPA Lombardia network, a comprehensive environmental monitoring system that has been operational for over 58 years, described by [4,5]. As of 2026, the network comprises 490 air quality stations strategically distributed across the Po river basin [5,6]. While the network monitors daily or hourly emissions for a diverse range of pollutants (including O_3_, CO, SO_2_ and heavy metals), this research is focused on four key pollutants which are common to the CAMS European reanalysis products: NO_*x*_, NO_2_, PM_2.5_ and PM_10_. Table 1, adapted from [4], shows the pollutants, frequency of measurements and number of stations.

These raw measurements from the in situ stations serve as the target labels for model training. ARPA concentrations appear only as response values and are never used to construct graph edges, OSM context or centrality.

### 3.3. OpenStreetMap Structural Context

To compute the contextually aware centrality measurements, the dataset incorporates urban structural data from OSM [66]. For each station, the context value is the count of OSM buildings within a 1 km buffer. Edges intersecting more than 1000 building features are excluded in the pre-defined graph refinement step. By identifying edges that intersect these physical features, the framework can further refine the graph topology, ensuring that centrality metrics like APAM or Eigendata reflect real-world functional significance rather than plain geometric connectivity [37].

### 3.4. Data Preprocessing

ARPA values coded as −9999 (the network’s missing-value sentinel) and non-numeric entries were set to missing. Daily targets were arithmetic means of the readings available within each day, aligning hourly CAMS and ARPA series to a common daily resolution. No statistical outlier deletion, temporal imputation or forward fill of missing observations was performed; the bilinear interpolation described above applies only to mapping CAMS grid values onto station coordinates. Each model used complete-case rows for its required target and predictors, without filling values from training or holdout periods. Table 2 reports target availability before model-specific complete-case filtering.

**Figure 4:**
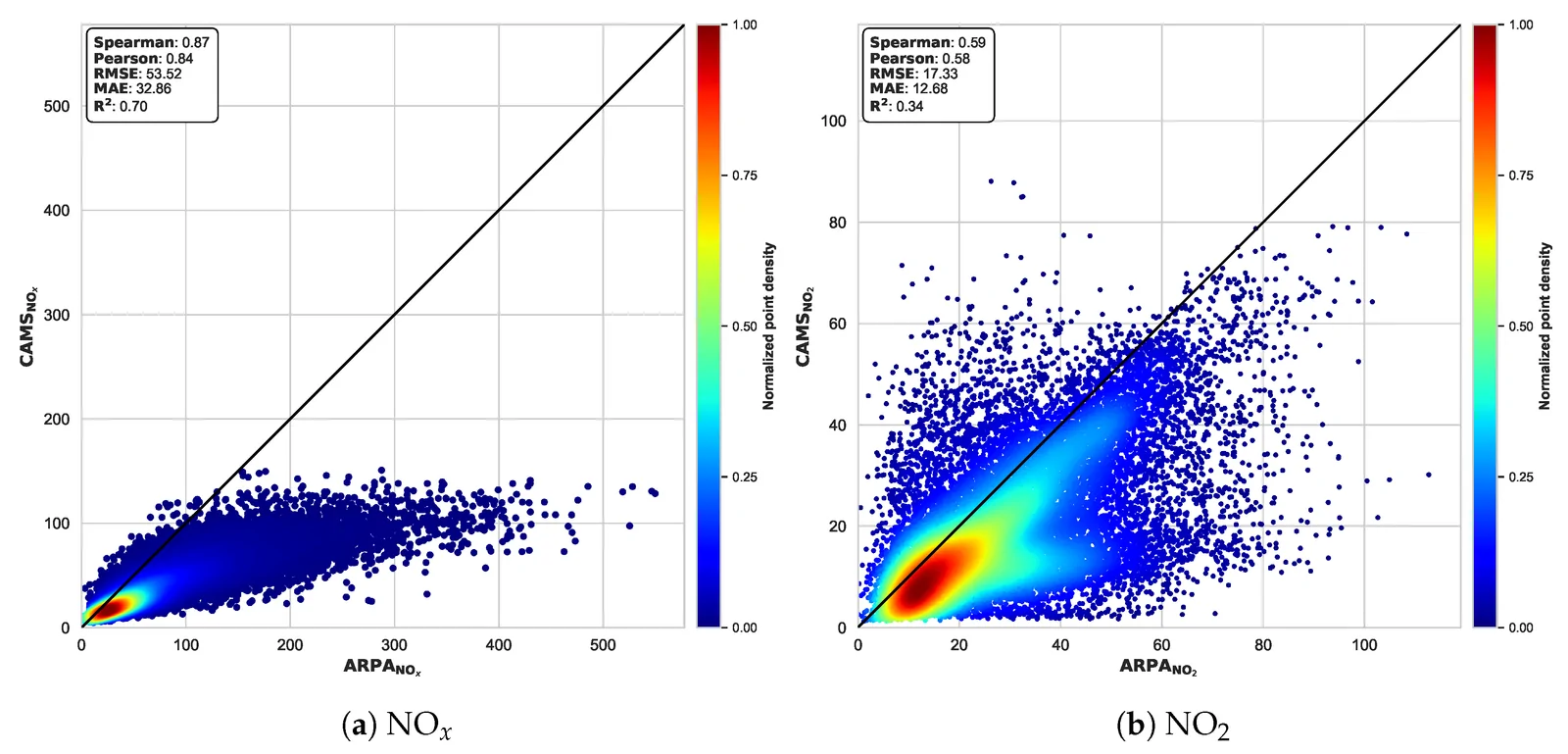

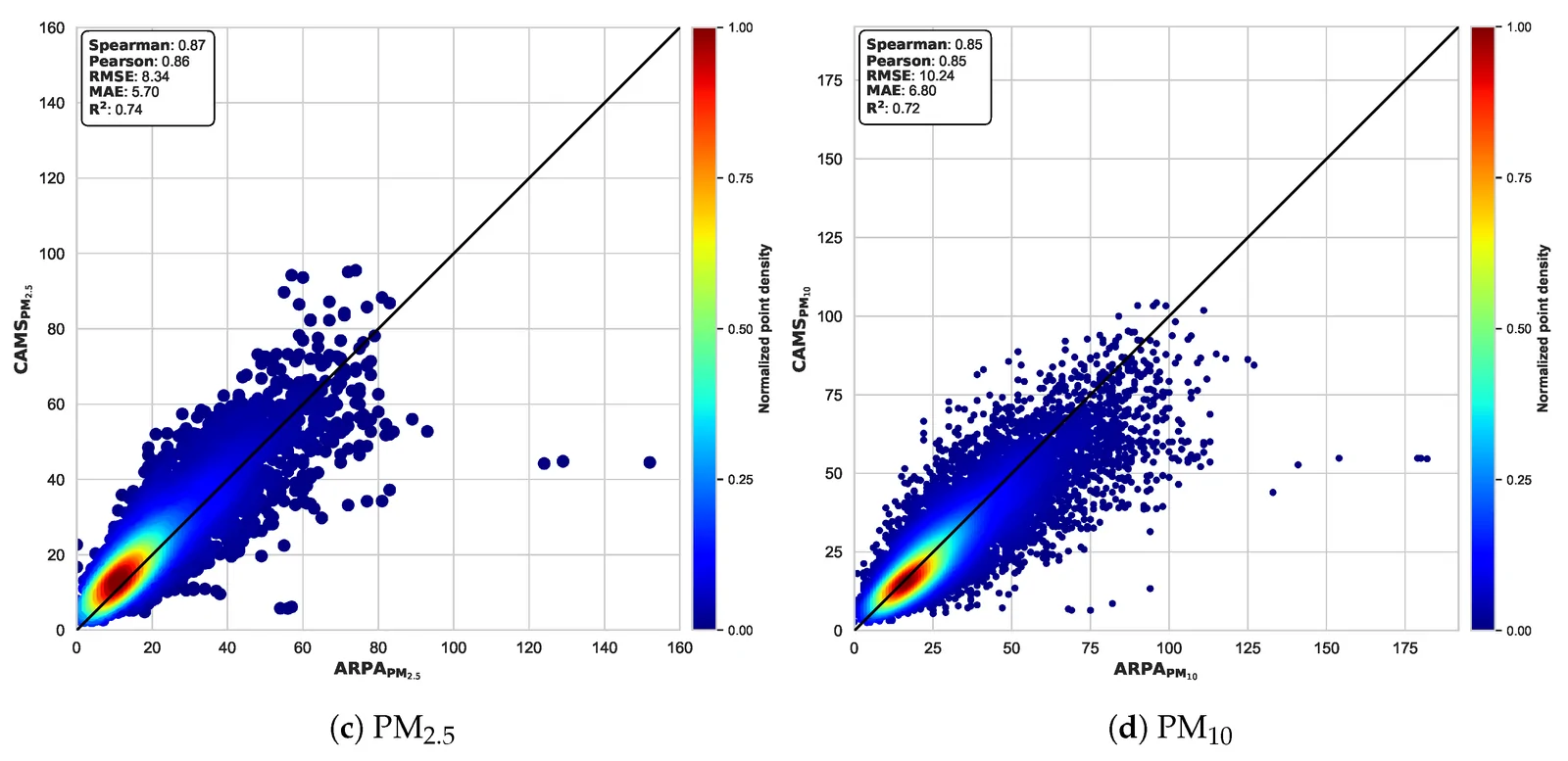
Station-day agreement between CAMS reanalysis estimates and available ARPA observations during 2018 to 2021 for NO_*x*_ (**a**), NO_2_ (**b**), PM_2.5_ (**c**) and PM_10_ (**d**). The diagonal is the 1:1 reference line; points are coloured by normalised scatter density. Panels annotate sample size *n*, Spearman and Pearson correlations, RMSE, MAE and $R^{2}$; both axes are in μg m^−3^.

Figure 4 shows station-day scatter plots of CAMS reanalysis estimates against ARPA observations for the 2018 to 2021 period; each panel annotates sample size *n*, Spearman and Pearson correlations, RMSE, MAE and one-to-one $R^{2}$. Pearson correlations were 0.84, 0.58, 0.86 and 0.85 for NO_*x*_, NO_2_, PM_2.5_ and PM_10_, respectively, indicating that CAMS tracks the observed temporal and spatial variability with moderate-to-strong linear association across all pollutants. However, corresponding one-to-one $R^{2}$ values of −0.823, 0.128, 0.201 and 0.246 reveal that association does not imply agreement: a high Pearson correlation is compatible with a large systematic offset. Mean bias (MB = mean of CAMS − ARPA over all paired station-days, computed from the data underlying Figure 4 but not annotated in the panels) was −45.40, −6.47, −0.25 and −4.62μg m^−3^ for NO_*x*_, NO_2_, PM_2.5_ and PM_10_, respectively. NO_*x*_ presents the most pronounced mismatch: the strongly negative one-to-one $R^{2}$ together with a mean bias of −45.40μg m^−3^ indicate that CAMS consistently and severely underestimates near-source NO_*x*_ peaks that individual stations capture but the 10 km ensemble cannot resolve. NO_2_ shows weaker correlation and a smaller yet still negative mean bias, suggesting both scatter and underestimation. For both PM fractions, near-zero mean biases coexist with one-to-one $R^{2}$ below 0.25, pointing to day-to-day and station-level scatter rather than a global offset; the visible upper-tail divergence from the 1:1 line reflects episodic accumulation events that the CAMS ensemble largely smooths away.

**Figure 5:**
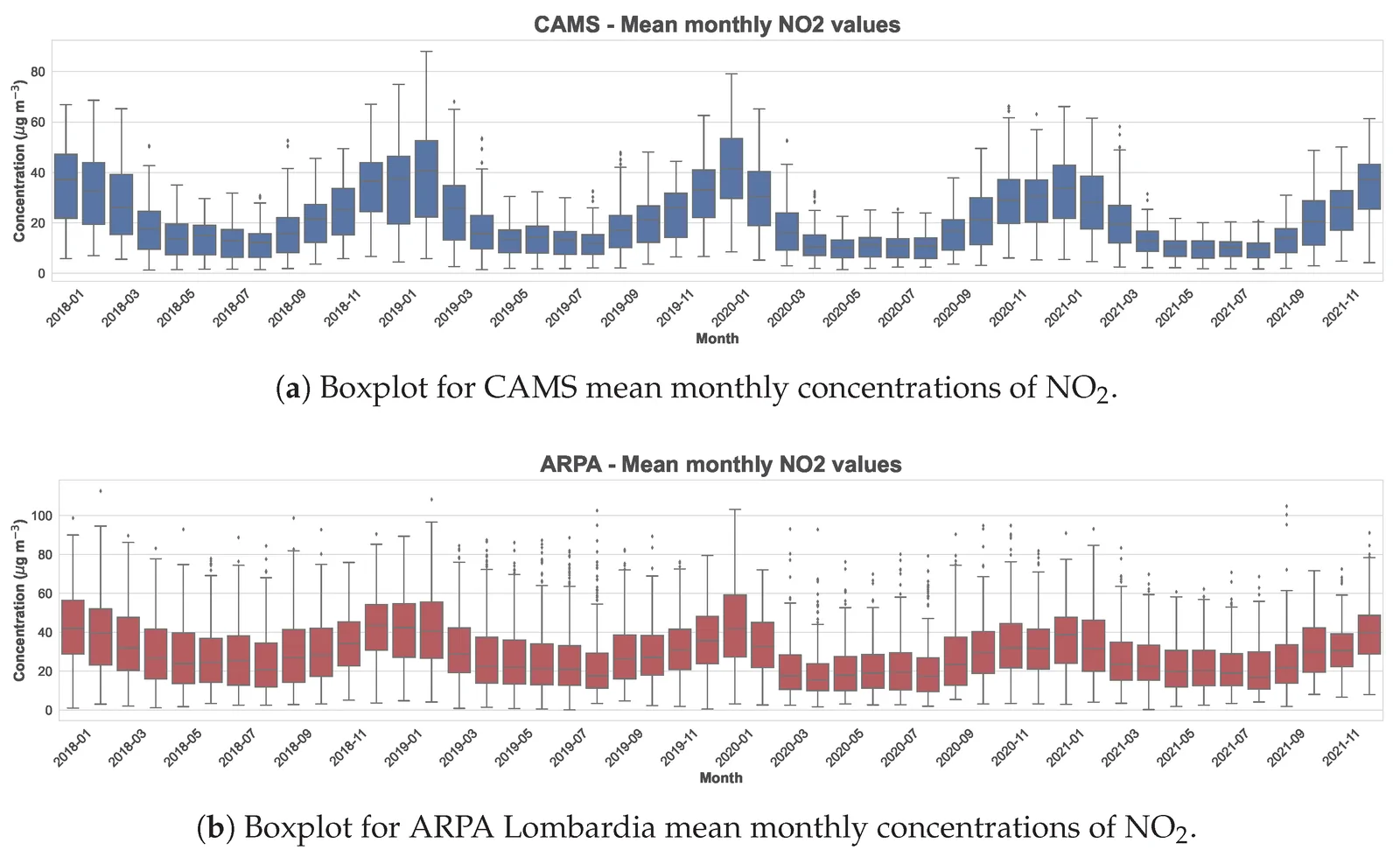
Mean monthly values of NO_2_ concentrations for CAMS (**a**) and ARPA (**b**) expressed in μg m^−3^. Boxes show the interquartile range, centre lines show medians, and whiskers extend to 1.5 IQR; each panel uses a uniform fill colour. Points beyond the whiskers are retained daily values; the wider spread in ARPA reflects local emission spikes and heating episodes captured by in situ monitoring stations but smoothed in the CAMS assimilated reanalysis. No statistical outlier deletion was applied.

In addition, we have investigated data seasonality for all of the four pollutants both in the case of in situ measurements and CAMS European reanalysis products. Consistent trends were present in both datasets, with pollutant values rising in the winter and falling in the summer. Figure 5 shows the corresponding trends between the mean monthly values in μg m^−3^ from both the CAMS European reanalysis products Figure 5a and ARPA Lombardia in situ measurements Figure 5b for NO_2_.

### 3.5. Graph Construction

Our approach towards building connectivity, similarly to the one proposed in [37], expresses both functional connectivity through K-Nearest neighbours (KNN) clustering based on node properties and spatial connectivity using a distance band heuristic, relying on pre-defined thresholds. The outcome of this connectivity is a multiplex graph network that represents the in situ measurement stations.

Optionally, the framework can further refine these graphs by eliminating redundant connections by employing minimum spanning tree (MST) algorithms such as Prim, Kruskal or Boruvka to completely drop redundant edges [67]. Another elective feature represents that the edges that intersect man-made structures can also be removed if certain feature type and threshold are specified. This step is performed by pooling and analysing the OpenStreetMap [66] features such as buildings or road networks that are intersecting with the edges, removing those edges above a specified threshold.

Distance-band thresholds of 1000 m and 20,000 m were pre-specified as a local structural scale and a broader sensitivity scale. The primary scale was 1000 m for NO_*x*_ and NO_2_ (short-lived, locally dominated gases) and 20,000 m for PM_2.5_ and PM_10_ (longer-lived particulate matter with regional transport). These choices are structural sensitivities rather than validated plume lengths or atmospheric transport distances; additional thresholds (5000 m and 10,000 m) appear in the sensitivity grid.

The node context vector is populated from OSM building counts within the configurable spatial radius, as described in Section 3.3. ARPA concentration measurements serve solely as daily response labels and are not used as node attributes or edge weights. To align them with the graph’s periodicity, available within-day readings are averaged to a single daily value per station.

All of the centrality measurements described in Section 2.1 can then be computed by the framework. Each configuration of the multiplex graph can be serialised as hive-partitioned GeoParquet data. Furthermore, CAMS data is spatially coregistered with the multiplex network graphs through bilinear interpolation at each station’s coordinates, while temporal alignment is performed using each observation window. Table 3 summarises the pollutant-specific OSM-refined graphs used in the primary analysis.

**Figure 6:**
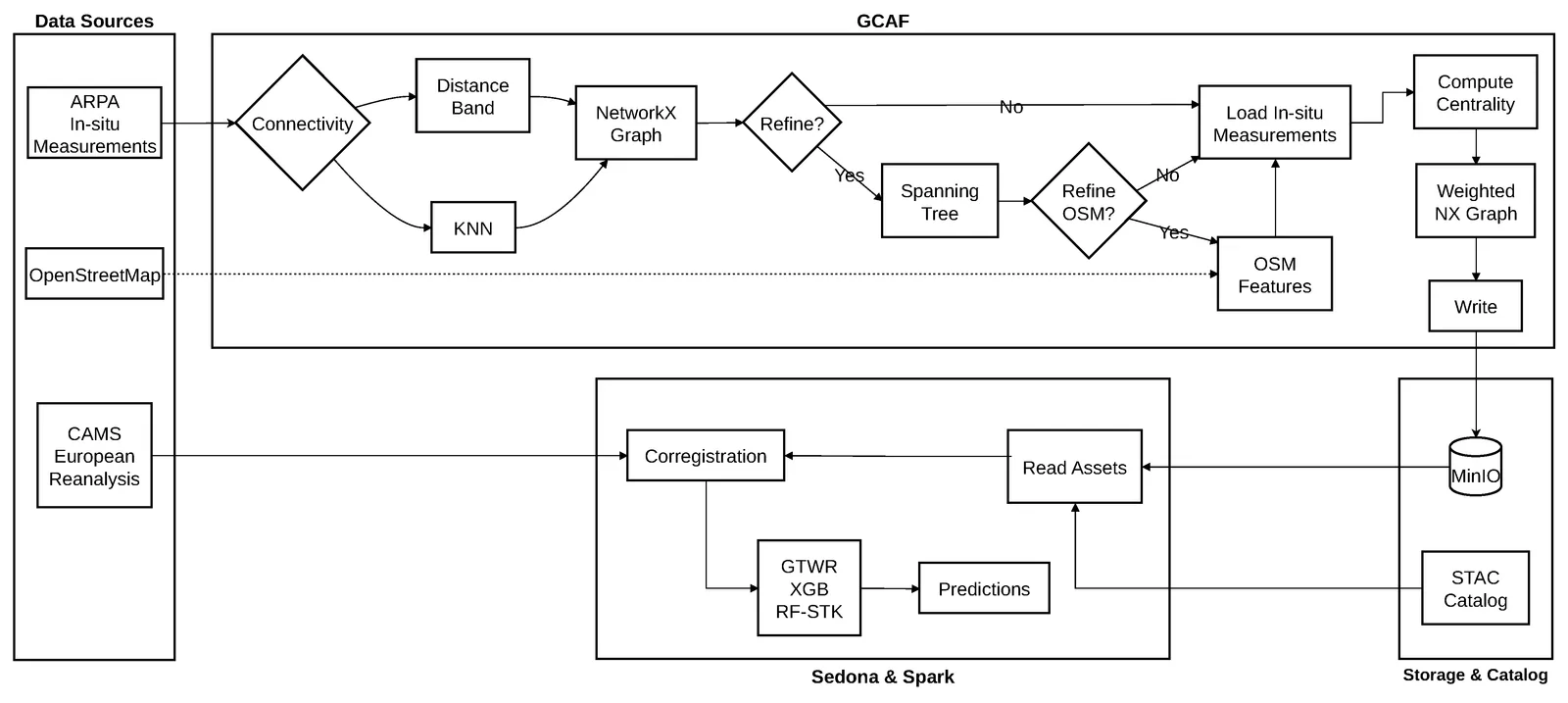
Workflow from data sources through connectivity, graph refinement, centrality, feature assembly and model training under blocked validation. Dashed arrows indicate the optional minimum spanning tree and OSM-refinement branches (Yes/No).

Three distinct models can be trained using this data: GTWR, XGBoost and RF-STK. Acting as proxies for structural urban context, the centrality measurements are added to the model as additional independent variables when the feature set includes them. The complete workflow is visually illustrated in Figure 6.

The experimental pipeline uses Apache Spark 4.0.1 [68] with Apache Sedona 1.8.1 [69] for distributed geospatial joins and GeoParquet for columnar storage.

### 3.6. Centrality Measures and Context-Aware Centrality

Classical centralities (PageRank, Betweenness, Eigenvector) were calculated on unweighted adjacency graphs. When minimum spanning trees were used, edge costs were physical station distance only. The primary context-aware feature was APA, with APAM and Eigendata retained as sensitivity analyses. For APA, APAM and Eigendata, the data vector *D* is the OSM building count within 1 km of each station. Pollutant measurements are not used to compute centrality features.

### 3.7. Machine Learning Models

We evaluate three distinct modelling approaches to capture different aspects of the spatial and graph-theoretic data, namely:

1. Geo-temporally weighted regression (GTWR) [32]: At each evaluation point, neighbours in space and time receive weights from a separable bisquare kernel. Spatial and temporal bandwidths were fixed a priori at 75 km and 45 days (regularization 0.25; at most 5000 training rows). Automatic bandwidth selection on the evaluation set was disabled.
2. XGBoost [33]: Configured with tree depth 8, 200 estimators and learning rate 0.05. Regularization uses min-child weight 2.0 and $\ell_{1}$/$\ell_{2}$ penalties (0.1/1.5) on leaf weights, with row and column subsampling 0.9/0.85.
3. RF-STK [34]: The random forest builds an ensemble of 100 trees (maximum depth 12, 64 bins, one-third feature subsampling, at least two observations per leaf). Residual spatio-temporal kriging uses cross-fitted training residuals only; holdout targets are never used to estimate the residual field.

The hyperparameters used for each model are summarised in Table 4. All values were specified before holdout evaluation; there was no tuning on station, temporal or combined holdouts.

Similar strategies for choosing hyperparameters are present in [61], where the authors suggest that optimal model performance is reached when the learning rate and maximum depth are tuned to minimize error metrics while maintaining stability across test sets. Furthermore, similar constraints for tree-based models such as fixed ensemble sizes and learning rates are discussed by [60] as a means to stabilize model training.

### 3.8. Validation and Experimental Design

Predictors are grouped into feature settings that form an ablation ladder, namely: centrality_only; cams_only; cams_spatial (CAMS and coordinates); cams_temporal (CAMS and time); cams_spatiotemporal (CAMS, coordinates and time); full (CAMS and centrality); and full_spatiotemporal (CAMS, coordinates, time and centrality).

The primary contrast keeps model, split, hyperparameters, CAMS, coordinates and time fixed and adds only OSM-context APA at the pollutant-specific primary scale (1000 m for NO_*x*_/NO_2_; 20,000 m for PM_2.5_/PM_10_). These feature sets are summarised in Table 5.

The experiment is comprised of 1080 total runs, out of which there are: 528 station-holdout runs, 528 temporal-holdout runs and 24 combined primary-contrast runs. Station holdout assigns complete stations to a fixed 20% test set. Temporal holdout trains on 2018 to 2020 and tests on 2021. The combined tests train before 2021 at non-held stations and evaluates 2021 at held stations. Primary effects are paired differences $\Delta ={\mathrm{error}}_{\mathrm{APA}}-{\mathrm{error}}_{\mathrm{baseline}}$ in RMSE and MAE, so negative values favour APA. We report station-cluster bootstrap 95% confidence intervals and two-sided station-level Wilcoxon signed-rank tests for MAE. $R^{2}$ is reported as a secondary metric alongside absolute errors in μg m^−3^.

It is noteworthy to mention that even though traditional current flow betweenness [43] and its resized variant proposed by [44] have been proven to be useful in localised urban contexts, they are computationally prohibitive at the scale of the graphs generated by the Graph Centrality Analysis Framework (GCAF) workflow and are therefore omitted in favour of more scalable centrality measurements.

## 4. Results

This section shows the predictive performance across the experimental grid and evaluates the contribution of graph-derived centrality features under blocked holdouts. We first analyse node influence patterns produced by the centrality measurements, then compare the performance of GTWR, XGBoost and RF-STK across the four pollutants, thresholds and feature-set configurations under station holdout. Lastly, we report the primary APA paired contrast and feature attribution for the best-performing models.

### 4.1. Identifying Node Influence Using Centrality Measurements

To evaluate whether centrality encodes meaningful local influence, we visualised the spatial distribution of node centrality values for a subset of configurations.

**Figure 7:**
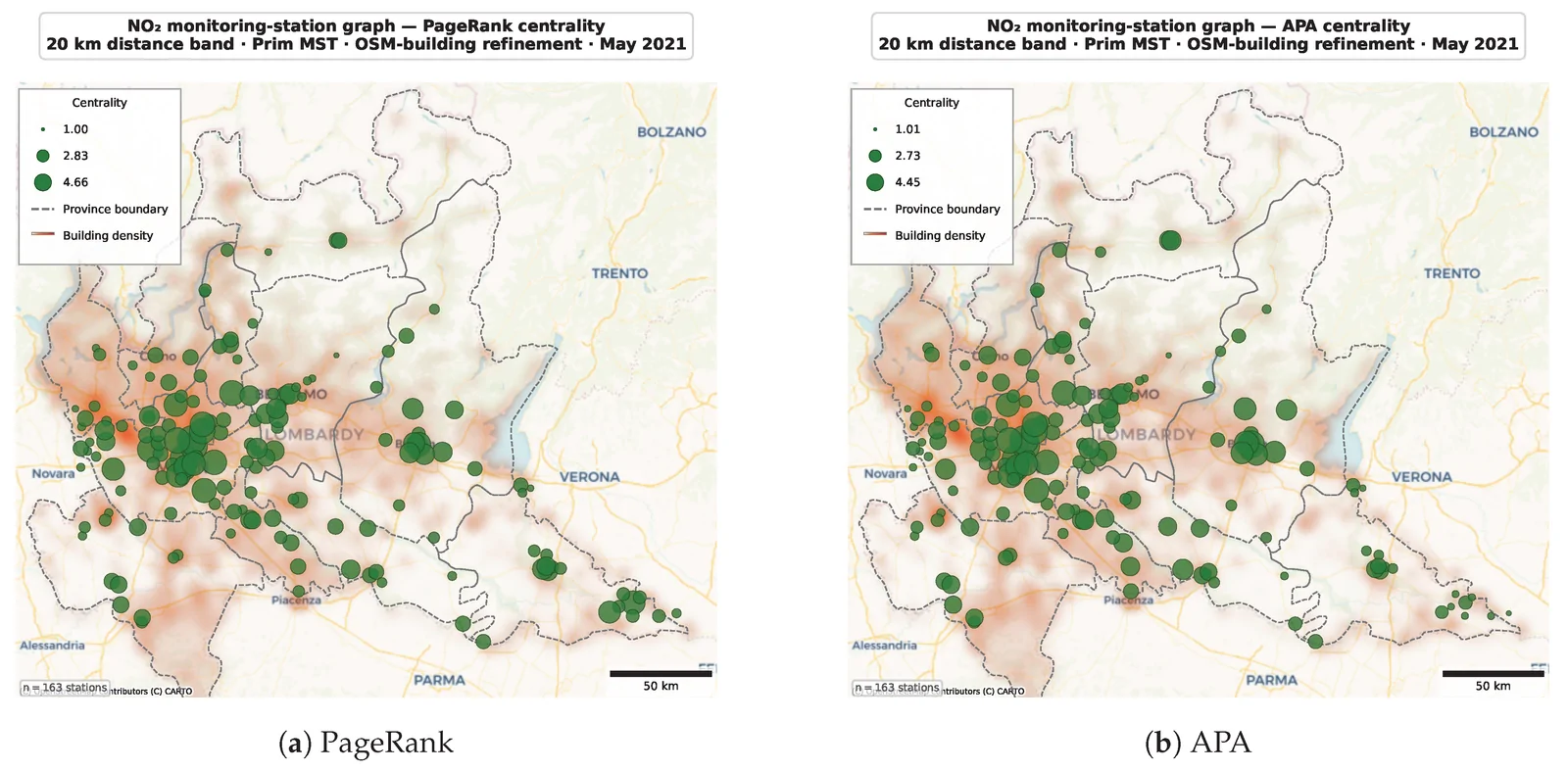

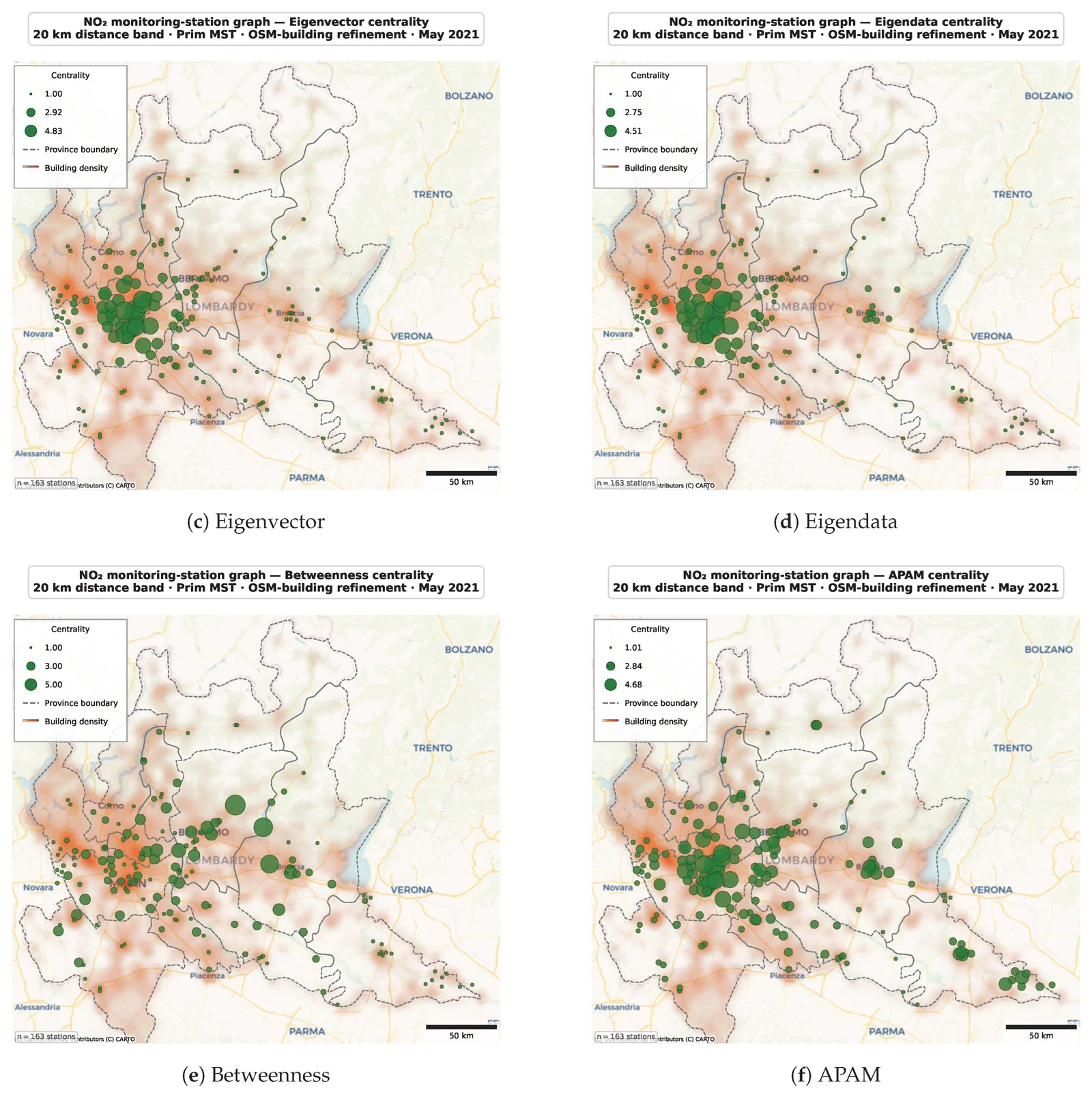
Node influence for NO_2_ stations using the six centrality measurements on the 20 km distance-band OSM-refined graph (163 stations, Prim MST, May 2021 snapshot): (**a**) PageRank; (**b**) Adapted PageRank Algorithm (APA); (**c**) Eigenvector; (**d**) Eigendata; (**e**) Betweenness and (**f**) Adapted PageRank Algorithm Modified. Coloured points represent the ARPA monitoring stations; symbol size represent the attributed centrality scores. The warm-toned overlay shows the OSM building density.

Figure 7 illustrates representative NO_2_ influence maps across six centrality measurements, computed on the broader 20 km distance-band graph (163 stations, Prim minimum spanning tree, OSM-building refinement, May 2021 snapshot) to give a region-wide view of the relative rankings.

The resulting maps show a clear non-uniform structure, with higher influence concentrated in dense urban settings, while peripheral areas exhibit systematically lower influence. Traditional measurements (PageRank Figure 7a, Betweenness Figure 7e, Eigenvector Figure 7c) and urban-context adapted variants (APA Figure 7b, APAM Figure 7f, Eigendata Figure 7d) produce consistent global patterns, while adapted variants generally show sharper local contrasts in context-rich urban zones, indicating improved sensitivity to functional structure beyond pure topology.

### 4.2. Evaluating Model Performance

To evaluate the performance of the models trained for each configuration, we use the following metrics: $R^{2}$ and adjusted $R^{2}$ to assess the proportion of variance captured by the model, Root Mean Square Error (RMSE) and Mean Square Error (MSE) to monitor prediction outliers and Mean Absolute Error (MAE) and Mean Absolute Percentage Error (MAPE) to assess the distance of the observations to the predictions in both absolute units (μg m^−3^) and relative percentage.

Table 6 summarizes the best-performing configuration per model under the station holdout validation strategy.

Under station holdout, XGBoost reaches $R^{2}=0.66$ for NO_2_ using CAMS with explicit spatial and temporal predictors (RMSE =10.64, MAE =8.05). RF-STK attains a higher peak $R^{2}$ of 0.81 for NO_2_ with Betweenness alone; this result should be interpreted in light of RF-STK’s residual spatial kriging, which can interpolate towards held-out stations even when the random-forest stage sees a lean feature set. GTWR remains lower ($R^{2}=0.48$ at best), consistent with the more limited flexibility of fixed-bandwidth local linear fits. Across pollutants, NO_2_ and PM_10_ remain the most favourable targets, while absolute errors for NO_*x*_ remain substantially larger than for NO_2_ or particulate matter.

Furthermore, Table 7 shows the best-performing configurations by pollutant-model pair under station holdout.

For XGBoost, the strongest NO_2_ fit uses cams_spatiotemporal, while several particulate-matter and NO_*x*_ configurations favour full_spatiotemporal, indicating that centrality can help but is not uniformly required once CAMS, space and time are available. RF-STK often ranks lean feature sets highly under station holdout, again consistent with residual kriging carrying spatial structure. GTWR remains below the tree-based models across all four pollutants.

Table 8 shows the 10 best-performing configurations for each model in terms of $R^{2}$ under station holdout. A clear pattern is the comparatively strong performance of the models when predicting NO_2_ and PM_10_. Temporal holdout yields lower peak $R^{2}$ values (approximately 0.48 for the best XGBoost and RF-STK configurations), confirming that forward-year generalisation is harder than station holdout within the same multi-year window.

### 4.3. Comparing Adapted PageRank to CAMS Baseline Configurations

Leader boards alone do not isolate the incremental value of centrality. Table 9 reports the pre-specified XGBoost contrast that adds OSM-context APA to CAMS, coordinates and time at the pollutant-specific graph scale.

No pollutant improved consistently across all three holdouts. Station-holdout PM_2.5_ showed small reductions in both errors with confidence intervals below zero, although the station-level Wilcoxon test was not significant (p=0.109, seven stations). Temporal PM_2.5_ had a small MAE reduction (p=0.044), while its RMSE interval included zero. The combined NO_*x*_ interval favoured APA, but only three held stations were available. The combined NO_2_ test indicated degradation.

Across matched full-spatiotemporal comparisons in the wider grid, APA improved 28 of 60 cells (median ΔRMSE near zero), while adding CAMS to centrality-only models reduced RMSE in 253 of 288 comparisons (median −2.635μg m^−3^), confirming that CAMS remains the dominant transferable information source.

A secondary sensitivity finding is temporal PM_10_ with RF-STK: APA reduced RMSE by 0.356 (95% CI −0.477 to −0.227) and MAE by 0.215μg m^−3^ (Wilcoxon $p=6.16\times 10^{-6}$). This effect persisted among 19 known-never-assimilated stations (ΔRMSE =−0.368μg m^−3^, p=0.0053 for MAE).

### 4.4. Importance of Features

To quantify how important each feature is within fitted models, we employ SHapley Additive exPlanations (SHAP) values [70]. As a game theory concept, SHAP values provide an importance score for every individual input feature by computing its average marginal contribution to the model’s output, taken over all possible subsets of features in which it can appear [70].

**Figure 8:**
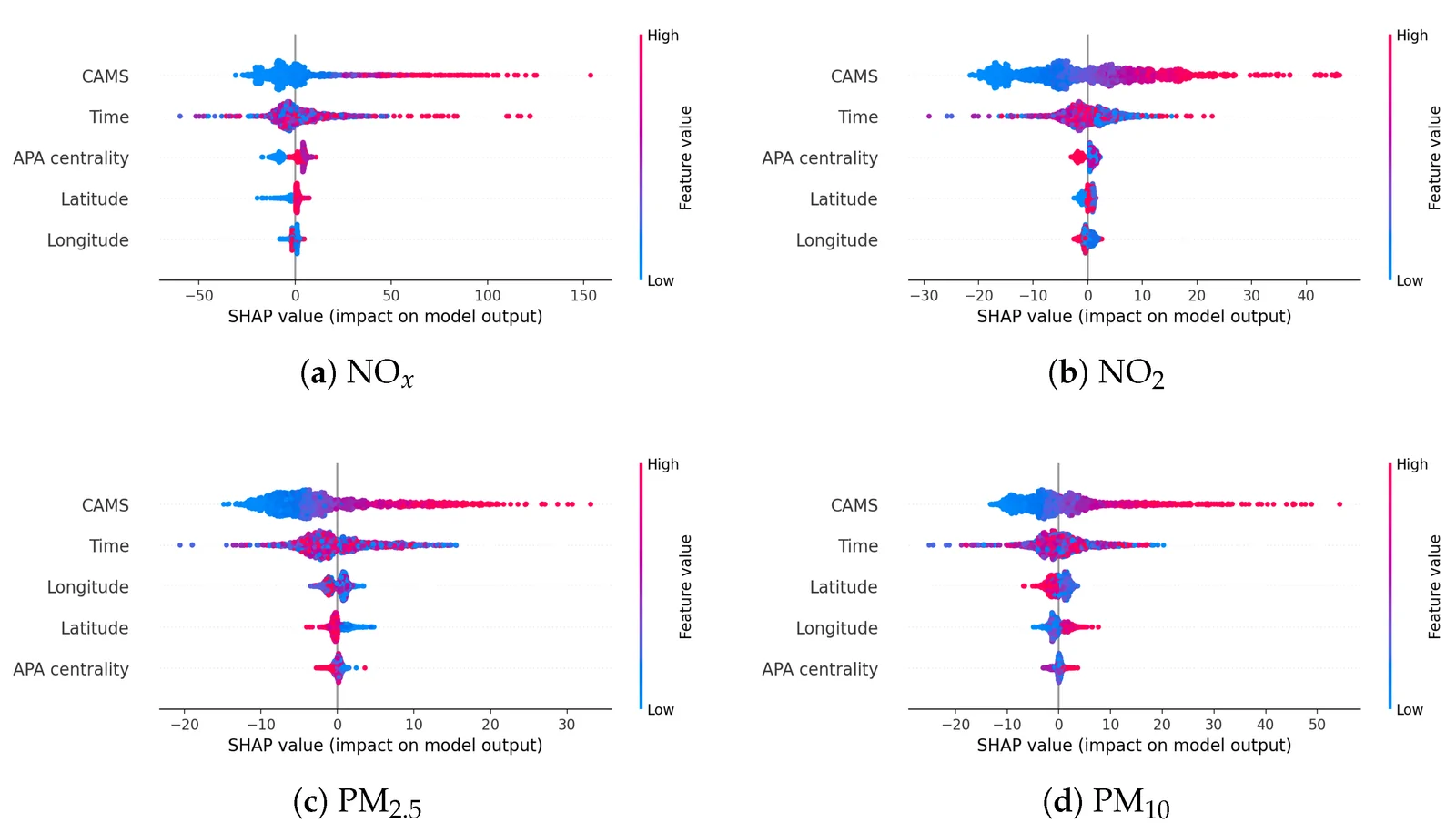
SHAP attribution distributions for station-holdout XGBoost models fitted with CAMS, coordinates, time, and APA centrality. Horizontal position gives the signed contribution to the model prediction (micrograms per cubic metre), colour encodes the corresponding feature value (blue low, red high), and each point is one held-out station-day (up to 2000 sampled from the validation split). Graph scale is 1 km for NO_*x*_ and NO_2_ and 20 km for PM_2.5_ and PM_10_. SHAP describes model attribution and does not imply causality.

Figure 8 summarises station-holdout SHAP attributions for the primary XGBoost configuration in each pollutant: CAMS, coordinates, time, and target-independent APA centrality at the pre-specified graph scale (1 km for NO_*x*_ and NO_2_; 20 km for PM_2.5_ and PM_10_). SHAP describes model attribution rather than causality; held-out ablation (Section 4.3) establishes incremental predictive value first. Because predictors can be correlated, attribution may be shared or redistributed among them, and mean absolute SHAP rank should not be interpreted as an independent effect size.

Feature-level interpretation is consistent with the ablation results: CAMS is the largest contributor in every panel, and time ranks second across all four pollutants. APA centrality is pollutant-dependent: it ranks third for NO_*x*_ and NO_2_ (mean |SHAP| of 5.2 and 1.2 μg m^−3^, respectively), but falls below the coordinate features for PM_2.5_ and PM_10_, where it is the smallest contributor (0.36 and 0.49 μg m^−3^). These attributions describe how each fitted model uses available predictors on held-out station-days; they do not establish that centrality has an independent causal effect.

## 5. Discussion

The results presented in Section 4 demonstrate that the GCAF framework [27] can support station-level correction of CAMS reanalysis products by integrating graph-theoretic structural information with in situ observations, but that the incremental value of centrality is smaller and more conditional than suggested by earlier random-split experiments. Under station holdout, XGBoost with CAMS, space and time reaches $R^{2}$ up to 0.66 for NO_2_, while RF-STK can achieve higher peak scores when residual spatial kriging is active. Adding OSM-context APA to an otherwise identical XGBoost baseline produces small, pollutant-dependent changes rather than a uniform lift.

The dominance of CAMS across the ablation ladder is expected as the reanalysis already integrates emissions, meteorology, chemistry, transport modelling and observations. Statistical learning can correct the station-level discrepancy. The station-day agreement results also show why correlation alone is insufficient: NO_*x*_ had moderate correlation but substantial negative bias and poor one-to-one agreement.

Furthermore, the findings show a divergence in model behaviour based on the pollutant type. NO_*x*_ and NO_2_ configurations remain more sensitive to local graph structure at the 1000 m scale, while particulate-matter models more often favour broader connectivity. We interpret these patterns as structural sensitivities rather than demonstrated atmospheric transport distances.

Centrality and OSM building density do not represent wind direction, atmospheric stability, boundary-layer height, topography, traffic intensity, industrial sources or chemical transformation; their omission plausibly contributes to residual spatial dependence (Moran’s *I* was nominally significant in more than 80% of fitted configurations).

The feature importance analysis in Section 4.4 is consistent with this reading. SHAP values confirm that CAMS contributes most to model output across all four pollutants, with time ranking second and APA centrality showing the largest attributed contribution for NO_*x*_ and a progressively smaller one for NO_2_, PM_10_, and PM_2.5_. Spatial coordinates contribute less than time but exceed APA centrality for the particulate-matter panels.

The most centrality-specific finding in the corrected grid is model-specific. For temporal PM_10_, RF-STK with APA reduced RMSE and MAE in both the full evaluation set and the 19-station known-but-never-assimilated subset. Its magnitude was nevertheless modest relative to an RMSE of approximately 15μg m^−3^. Replication in other years or monitoring networks is needed before treating this as a general PM_10_ relationship.

From an architectural perspective, scalable and reproducible geospatial processing used the Apache Sedona [69] and Apache Spark [68] frameworks.

Future work should explore interaction features (e.g., centrality × cams_value), integrate dynamic traffic flow and weather data (wind speed and direction from ARPA meteorological stations [4]), and incorporate Digital Elevation Models to account for terrain elevation. Another research avenue is Current Flow Betweenness (CFB) [43] and Resized Current Flow Betweenness (RCFB) [44]. Although they were unfeasible here due to computational complexity, distributed approaches such as [71] could scale such centralities. Other models such as Light Gradient Boosting (LightGBM) [72], Support Vector Machines (SVMs) [73] or Artificial Neural Networks (ANNs) might also achieve good performance [60,61], and hyperparameter optimization frameworks could also be employed [61].

## 6. Conclusions

This study evaluated whether target-independent graph centrality improves station-level correction of CAMS NO_*x*_, NO_2_, PM_2.5_ and PM_10_ concentrations in Lombardy. CAMS provided substantial predictive information, while OSM-context APA centrality had small, incremental effects and did not yield a general improvement across the four pollutants.

Under station holdout, XGBoost reached $R^{2}$ up to 0.66 for NO_2_ with CAMS, coordinates and time. RF-STK often ranked highly when residual spatial kriging contributed, and the most centrality gain was the temporal PM_10_ RF-STK result. Smaller favourable effects for PM_2.5_ and the combined NO_*x*_ contrast identify settings worth further study, but their uncertainty and protocol dependence limit generalization.

The contributions of this work can be summarised as follows:

1. Extending the GCAF Framework: We extend the GCAF framework with a centrality pipeline integrating APA/APAM and Eigendata using OSM building density as the context vector, evaluated under station, temporal and combined holdout strategies.
2. Multiplex Graph Construction: We propose a method for generating station networks where edge weights are refined by physical urban features from OpenStreetMap, with characterised graph topology and pollutant-specific structural scales reported across centrality variants.
3. Structured Feature Evaluation: We design an incremental ablation over CAMS reanalysis, spatial coordinates, temporal information and graph centrality, accompanied by paired error contrasts and uncertainty estimates for each experimental configuration.
4. Empirical Validation: Through an evaluation of 1080 experimental configurations, we demonstrate that non-linear models remain competitive for station-level CAMS correction, with centrality contributing as a complementary, model-dependent covariate whose effect varies across pollutants and holdout strategies.

## Figures and Tables

**Table 1:** Air quality monitoring stations operated by ARPA Lombardia [4] used for this study.

Pollutant	Symbol	Frequency	Stations
Total nitrogen oxides	${\mathrm{NO}}_{x}$	Hourly	88
Nitrogen dioxide	${\mathrm{NO}}_{2}$	Hourly	88
Particulate matter 2.5	${\mathrm{PM}}_{2.5}$	Daily	10
Particulate matter 10	${\mathrm{PM}}_{10}$	Daily	64

**Table 2:** ARPA target availability before model-specific complete-case filtering. CAMS was available for all station-days shown.

Pollutant	Stations	Possible Days	Complete Days	Missing Target (%)
NO_*x*_	84	238,143	117,418	50.69
NO_2_	85	238,143	118,853	50.09
PM_2.5_	35	58,440	44,574	23.73
PM_10_	64	128,568	87,948	31.59

**Table 3:** Pollutant-specific characteristics of the OSM-refined graphs used in the primary analysis. Threshold is the station distance-band radius; observed stations are nodes with target observations during 2018 to 2021. Mean degree and counts are dimensionless.

Pollutant	Threshold (km)	Graph Nodes	Observed Stations	Edges	Components	Isolates	Mean Degree
NO_*x*_	1	17	13	8	9	2	0.94
NO_2_	1	17	13	8	9	2	0.94
PM_2.5_	20	40	35	28	12	6	1.40
PM_10_	20	88	64	78	10	2	1.77

**Table 4:** Hyperparameters chosen for the models.

Model	Hyperparameter	Value
GTWR	Bandwidth selection	Fixed 75 km spatial/45 d temporal (no holdout tuning)
	Kernel	Bisquare
	Kernel regularization	0.25
	Max. training rows for neighbourhoods	5000
XGBoost	Max. depth/estimators	8/200
	Learning rate	0.05
	Subsample/col. sample	0.9/0.85
	Regularization	min. child weight: 2.0; $\ell_{2}$: 1.5; $\ell_{1}$: 0.1
RF-STK	Trees/max. depth	100/12
	Max. bins	64
	Feature subsampling	One-third of inputs per split
	Min. instances per leaf	2
	Residual kriging	Cross-fitted training residuals only

**Table 5:** Feature sets used for the experiments.

Configuration	Features	Description
centrality_only	centrality_value	Graph node centrality only.
cams_only	cams_value	CAMS reanalysis data only.
cams_spatial	cams_value, x, y	CAMS plus coordinates.
cams_temporal	cams_value, period_start_unix	CAMS plus time.
cams_spatiotemporal	cams_value, x, y, period_start_unix	CAMS plus spatial and temporal features.
full	centrality_value, cams_value	Graph node centrality values + CAMS data.
full_spatiotemporal	centrality_value, cams_value	Centrality, CAMS plus spatial
	x, y, period_start_unix	and temporal features.

**Table 6:** Best-performing configuration per model under station holdout. For feature sets that do not include centrality (e.g., cams_spatiotemporal), the Centrality column identifies the graph partition used for station alignment only.

Model	Pol.	Feature Set	Thr.	Feat.	Centrality	$R^{2}$	Adj. $R^{2}$	RMSE	MSE	MAE	MAPE
XGBoost	NO_2_	cams_spatiotemporal	1000	1000	-	0.6610	0.6607	10.6387	113.1821	8.0502	48.3937
RF-STK	NO_2_	centrality_only	1000	1000	Betweenness	0.8109	0.8109	7.9452	63.1256	6.1633	32.6322
GTWR	NO_2_	full	1000	1000	Eigendata	0.4803	0.4800	13.1723	173.5089	10.0437	68.5041

**Table 7:** Best configuration per pollutant and model under station holdout.

Pollutant	Model	Feature Set	Thr.	Feat.	Centrality	$R^{2}$	Adj. $R^{2}$	RMSE	MSE	MAE	MAPE
NO_*x*_	XGBoost	full_spatiotemporal	1000	1000	Eigendata	0.6023	0.6018	28.9586	838.5989	20.8024	96.7103
NO_*x*_	RF-STK	cams_only	1000	1000	-	0.7321	0.7321	23.7649	564.7700	15.7373	59.9753
NO_*x*_	GTWR	full_spatiotemporal	1000	1000	Eigendata	0.4018	0.4011	35.5146	1261.2859	22.6503	87.1008
NO_2_	XGBoost	cams_spatiotemporal	1000	1000	-	0.6610	0.6607	10.6387	113.1821	8.0502	48.3937
NO_2_	RF-STK	centrality_only	1000	1000	Betweenness	0.8109	0.8109	7.9452	63.1256	6.1633	32.6322
NO_2_	GTWR	full	1000	1000	Eigendata	0.4803	0.4800	13.1723	173.5089	10.0437	68.5041
PM_10_	XGBoost	full_spatiotemporal	1000	1000	Eigendata	0.6280	0.6273	10.7346	115.2313	7.8552	42.4895
PM_10_	RF-STK	cams_temporal	20,000	1000	-	0.7723	0.7722	8.3691	70.0410	5.9630	38.6038
PM_10_	GTWR	cams_temporal	1000	1000	-	0.4194	0.4190	13.4104	179.8401	9.5368	52.6166
PM_2.5_	XGBoost	full_spatiotemporal	20,000	1000	PageRank	0.5839	0.5835	9.8212	96.4563	6.8168	47.8650
PM_2.5_	RF-STK	centrality_only	1000	1000	PageRank	0.7286	0.7284	8.1540	66.4884	6.1669	66.4385
PM_2.5_	GTWR	cams_temporal	1000	1000	-	0.4122	0.4113	12.0011	144.0259	8.6269	113.9281

**Table 8:** Top 10 configurations for each model.

Model	Pollutant	Feature Set	Thr.	Feat.	Centrality	$R^{2}$	Adj. $R^{2}$	RMSE	MSE	MAE	MAPE
XGBoost	NO_2_	cams_spatiotemporal	1000	1000	-	0.6610	0.6607	10.6387	113.1821	8.0502	48.3937
XGBoost	NO_2_	full_spatiotemporal	1000	1000	Betweenness	0.6609	0.6605	10.6404	113.2184	8.0482	49.8508
XGBoost	NO_2_	full_spatiotemporal	1000	1000	Eigenvector	0.6327	0.6323	11.0737	122.6268	8.5544	55.9301
XGBoost	NO_2_	full_spatiotemporal	1000	1000	APAM	0.6290	0.6286	11.1286	123.8454	8.5950	56.1402
XGBoost	NO_2_	full_spatiotemporal	1000	1000	PageRank	0.6286	0.6282	11.1355	123.9984	8.6058	56.1088
XGBoost	PM_10_	full_spatiotemporal	1000	1000	Eigendata	0.6280	0.6273	10.7346	115.2313	7.8552	42.4895
XGBoost	PM_10_	full_spatiotemporal	1000	1000	APA	0.6243	0.6237	10.7871	116.3610	7.9364	43.3256
XGBoost	PM_10_	full_spatiotemporal	1000	1000	PageRank	0.6233	0.6226	10.8026	116.6955	8.1415	47.0043
XGBoost	PM_10_	full_spatiotemporal	1000	1000	Betweenness	0.6233	0.6226	10.8026	116.6955	8.1415	47.0043
XGBoost	PM_10_	full_spatiotemporal	1000	1000	Eigenvector	0.6233	0.6226	10.8026	116.6955	8.1415	47.0043
RF-STK	NO_2_	centrality_only	1000	1000	Betweenness	0.8109	0.8109	7.9452	63.1256	6.1633	32.6322
RF-STK	PM_10_	cams_temporal	20,000	1000	-	0.7723	0.7722	8.3691	70.0410	5.9630	38.6038
RF-STK	PM_10_	cams_spatial	20,000	1000	-	0.7690	0.7689	8.4294	71.0543	6.0647	38.4989
RF-STK	PM_10_	centrality_only	1000	1000	PageRank	0.7684	0.7683	8.4701	71.7419	6.0276	29.9626
RF-STK	PM_10_	centrality_only	1000	1000	Eigenvector	0.7684	0.7683	8.4701	71.7419	6.0276	29.9626
RF-STK	PM_10_	centrality_only	1000	1000	APAM	0.7684	0.7683	8.4701	71.7419	6.0276	29.9626
RF-STK	PM_10_	centrality_only	1000	1000	Betweenness	0.7684	0.7683	8.4701	71.7419	6.0276	29.9626
RF-STK	PM_10_	full_spatiotemporal	20,000	1000	Eigendata	0.7631	0.7629	8.5359	72.8615	6.1207	38.6642
RF-STK	PM_10_	full_spatiotemporal	20,000	1000	PageRank	0.7625	0.7623	8.5466	73.0447	6.0897	38.9577
RF-STK	PM_10_	full_spatiotemporal	20,000	1000	APA	0.7617	0.7615	8.5611	73.2925	6.1016	38.8275
GTWR	NO_2_	full	1000	1000	Eigendata	0.4803	0.4800	13.1723	173.5089	10.0437	68.5041
GTWR	NO_2_	full_spatiotemporal	1000	1000	Eigendata	0.4684	0.4678	13.3218	177.4713	10.1663	68.5398
GTWR	NO_2_	cams_temporal	1000	1000	-	0.4615	0.4613	13.4080	179.7740	10.2692	68.7389
GTWR	NO_2_	cams_only	1000	1000	-	0.4605	0.4603	13.4212	180.1288	10.2344	68.5048
GTWR	NO_2_	full	1000	1000	Betweenness	0.4605	0.4602	13.4212	180.1278	10.2318	68.5056
GTWR	NO_2_	full	1000	1000	PageRank	0.4595	0.4592	13.4330	180.4465	10.2680	69.2056
GTWR	NO_2_	full	1000	1000	Eigenvector	0.4595	0.4592	13.4331	180.4472	10.2678	69.2016
GTWR	NO_2_	full	1000	1000	APAM	0.4595	0.4592	13.4331	180.4469	10.2680	69.2059
GTWR	NO_2_	full	1000	1000	APA	0.4550	0.4548	13.4888	181.9475	10.3434	71.5998
GTWR	NO_2_	cams_spatiotemporal	1000	1000	-	0.4510	0.4505	13.5384	183.2896	10.3346	68.0114

**Table 9:** Primary paired XGBoost contrast: OSM-context APA added to CAMS, coordinates and time. Values are ΔRMSE and ΔMAE in μg m^−3^ (95% station-cluster bootstrap CI); negative values favour APA. *p* is the two-sided station-level Wilcoxon signed-rank test for paired MAE.

Holdout	Pollutant	ΔRMSE (95% CI)	ΔMAE (95% CI)	*p*
Station	NO_*x*_	−2.17 (−6.69, 1.04)	−1.52 (−6.30, 2.06)	0.750
	NO_2_	2.06 (−0.10, 4.97)	2.03 (−0.11, 5.52)	0.500
	PM_2.5_	−0.20 (−0.39, −0.07)	−0.23 (−0.55, −0.003)	0.109
	PM_10_	−0.06 (−0.14, 0.03)	−0.06 (−0.15, 0.03)	0.216
Temporal	NO_*x*_	0.63 (0.07, 1.27)	0.59 (−0.09, 1.30)	0.244
	NO_2_	0.21 (−0.37, 0.62)	0.25 (−0.27, 0.65)	0.080
	PM_2.5_	−0.09 (−0.20, 0.04)	−0.15 (−0.28, −0.02)	0.044
	PM_10_	0.01 (−0.05, 0.05)	−0.01 (−0.07, 0.05)	0.984
Station-temporal	NO_*x*_	−7.51 (−19.69, −0.02)	−6.85 (−18.22, −0.01)	0.250
	NO_2_	2.31 (0.11, 5.36)	2.75 (0.22, 6.11)	0.250
	PM_2.5_	−0.35 (−1.13, 0.26)	−0.38 (−1.30, 0.35)	0.813
	PM_10_	0.18 (−0.08, 0.54)	0.17 (−0.07, 0.48)	0.340

## Data Availability

The source code utilised for the experiments in this study is publicly available online at https://doi.org/10.5281/zenodo.20346943 (accessed on 22 May 2026). The dataset containing graph data, in situ measurements, centrality measurements, raw CAMS data and STAC metadata is available at https://doi.org/10.5281/zenodo.20346296 (accessed on 22 May 2026). Analysis run manifests and derived result tables will accompany the final archived release.
